# Flexible and navigable suction access sheaths: balancing sheath and scope size for desired flows

**DOI:** 10.1111/bju.16865

**Published:** 2025-09-26

**Authors:** Richard Menzies‐Wilson, Jessica Williams, Thijs Ruiken, Candace Rhodes, Ben Turney

**Affiliations:** ^1^ Nuffield Department of Surgery University of Oxford Oxford UK; ^2^ Boston Scientific Corporation Marlborough MA USA

**Keywords:** flexible and navigable suction access sheaths (FANS), ureteroscope, intrarenal pressure (IRP), irrigation pressure, outflow resistance

## Abstract

**Objective:**

To study the effects of ureteroscope diameter, ‘flexible and navigable suction’ access sheaths (FANS) diameter, and irrigation pressure on intrarenal pressure (IRP) and irrigation fluid flow rates in *ex vivo* porcine kidneys; these benchtop data were compared against mathematical modelling results.

**Materials and Methods:**

Fresh *ex vivo* porcine kidneys and ureters were used. The tip of an 11/13‐ or 10/12‐F ClearPetra*®* FANS was placed within the renal pelvis. Either a 9.5‐F Boston Scientific LithoVue™, 7.5‐F PUSEN PU3033, or 6.3‐F HugeMed HU30M ureteroscope was inserted through the sheath. Irrigation pressures of 0, 50, 100, 150 and 200 mmHg were applied. No suction was applied. The corresponding steady state IRP and flow rate was recorded. This was replicated in three separate porcine kidneys. Benchtop data were compared to mathematical model predictions. The maximum flow rates that could be achieved with an IRP ≤30 mmHg were extrapolated.

**Results:**

The FANS and ureteroscope geometries affect outflow resistance. Lower outflow resistances, allow higher irrigation pressures and flow rates to be used whilst maintaining an IRP ≤30 mmHg (without suction). Whilst keeping IRP ≤30 mmHg with an 11/13‐F FANS: ~700 mmHg irrigation (~120 mL/min) could be used with a 6.3‐F ureteroscope; ~300 mmHg irrigation (flow rate ~65 mL/min) could be used with a 7.5‐F ureteroscope; ~100 mmHg irrigation (~15 mL/min) could be used with a 9.5‐F ureteroscope.

**Conclusions:**

Using a FANS in the renal pelvis potentially allows for higher irrigation pressures whilst maintaining a low IRP (without the need for suction). With intraoperative monitoring of IRP, irrigation pressure could be increased to achieve flow rates up to 24 times higher than those achievable with gravity irrigation (120 vs ~5 mL/min).

**Abbreviations**
FANS: flexible and navigable suction access sheath
IRP: intrarenal pressure
RESD: ratio of endoscope to sheath diameter
RIRS: retrograde intrarenal surgery
URS: ureteroscopy

## Introduction

During ureteroscopy (URS), urologists typically use irrigation fluid to maintain vision and create a working space within the renal collecting system. Increasing irrigation fluid pressure improves irrigation flow and thus operative vision [1]. However, higher irrigation fluid pressure can elevate the intrarenal pressure (IRP) within the renal collecting system [2]. Consequently, urologists limit irrigation pressure due to concerns of adverse effects from an excessively raised IRP. It is commonly thought that maintaining IRP below a threshold of 30 mmHg (40 cmH_2_O) can minimise pyelovenous backflow, which could minimise the risk of infectious complications [2, 3, 4]. However, IRP during flexible URS was recently shown to frequently exceed this threshold [5, 6].

The IRP (and irrigation flow) depends on three main factors: irrigation pressure, inflow resistance (this varies based on working channel size and tools in the working channel), and outflow resistance (the impedance encountered by irrigation fluid as it exits the kidney) [1]. Inflow and outflow resistances have a significant effect on IRP at any given irrigation pressure. Consequently, the maximum irrigation pressure that can be used whilst maintaining an IRP ≤30 mmHg varies considerably based on these factors [1].

The use of traditional (non‐flexible) ureteric access sheaths is understood to reduce outflow resistance, and so IRP, compared to not using an access sheath [6]. Traditional ureteric access sheaths are typically placed with the tip in the proximal ureter, just below the PUJ [7]. The PUJ acts as a functional valve, restricting fluid egress from the renal pelvis [8]. Using a flexible and navigable suction access sheath (FANS), the tip is navigated past the PUJ into the renal collecting system. This allows fluid to drain directly out of the patient through the sheath – bypassing the PUJ. This results in reduced ‘outflow resistance’ and lower IRPs compared to using a typical ureteric access sheath, even without applying suction. Consequently, when a FANS sheath is initially inserted the renal collecting system is often seen to collapse.

**Fig. 1:**
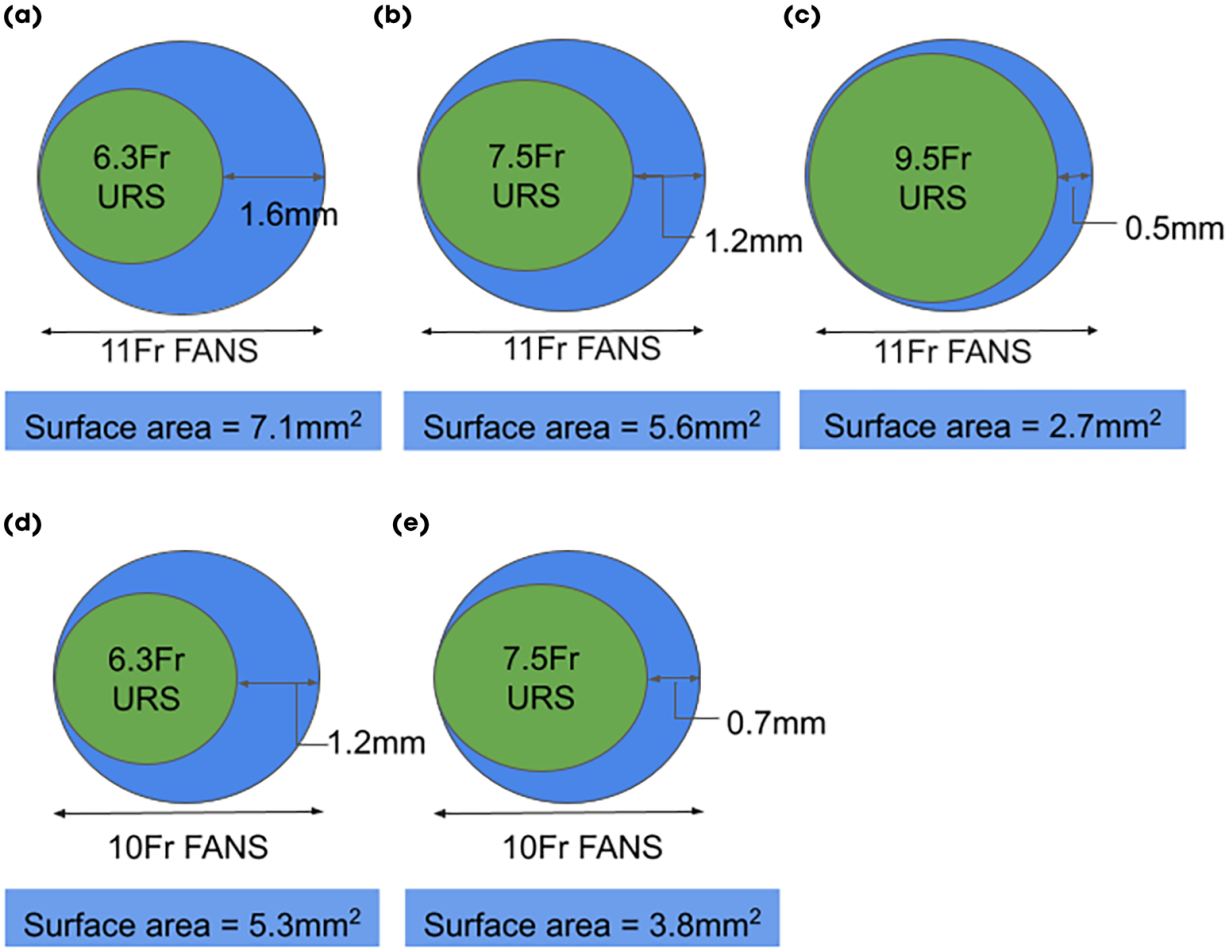
Theoretical comparison of cross‐sectional areas of two FANS sizes with different sized indwelling ureteroscopes. The blue boxes show the outflow cross sectional area between the FANS inner diameter and the ureteroscope outer diameter which determines the fluid outflow resistance. (**a**) 6.3‐F ureteroscope (green circle) in 11/13‐F FANS (blue circle); (**b**) 7.5‐F ureteroscope in 11/13‐F FANS; (**c**) 9.5‐F ureteroscope in 11/13‐F FANS; (**d**) 6.3‐F ureteroscope in 10/12‐F FANS; (**e**) 7.5‐F ureteroscope in 10/12‐F FANS.

In this scenario, outflow resistance is determined by the cross‐sectional area between the outer diameter of the ureteroscope and the inner diameter of the access sheath (Fig. 1). The larger the difference between the diameters of the ureteroscope and FANS, the larger the cross‐sectional area for fluid egress, the lower the outflow resistance, and lower the IRP. This should facilitate higher irrigation pressures (and flow rates) than typically used during URS whilst maintaining a low IRP.

To utilise FANS to maximum effect, it is essential for urologists to understand how to combine ureteroscope and FANS geometry to control the outflow resistance. With a lower outflow resistance, the surgeon can leverage the benefits of higher irrigant flow rates (improving vision, particle washout, and potentially allowing higher laser power to be used by mitigating the thermal dose) [1, 9, 10, 11]. However, the outflow resistance may be too low, requiring very high irrigation pressures, and flow rates, to distend the collecting system and create enough operative space. A minimum IRP needed to create operative space is poorly defined in the literature; however, baseline (non‐distended) kidney IRP has been reported between 10 and 16 mmHg by several clinical studies [12, 13]. Additionally, an *in vivo* porcine experiment reports that the ‘surgical field is narrow…with inadequate lighting’ at an IRP of 12.59–20.56 mmHg (the head‐high‐foot‐low position [H‐H‐F‐L] group at 50 mL/min) [8].

To better understand the effect of ureteroscope size, FANS diameter, and irrigation fluid pressure on IRP, we conducted URS simulated fluidics in *ex vivo* porcine kidney experiments. We quantified the IRPs and flow rates with various combinations of commonly used ureteroscopes and FANS. All data collected were with the FANS vent open and no suction applied, so as to reflect the functionality of most currently available FANS [14]. These incorporate vent designs that, when uncovered (as is typically the case), may function as an air off‐valve [15]. As a result, suction applied to the FANS may not influence IRP during the majority of the procedure. Instead, ambient air may be preferentially aspirated through the open vent [15].

These benchtop data were used to validate a theoretical mathematical physics‐based model to predict behaviours across a larger set of theoretical parameters.

## Materials and Methods

**Fig. 2:**
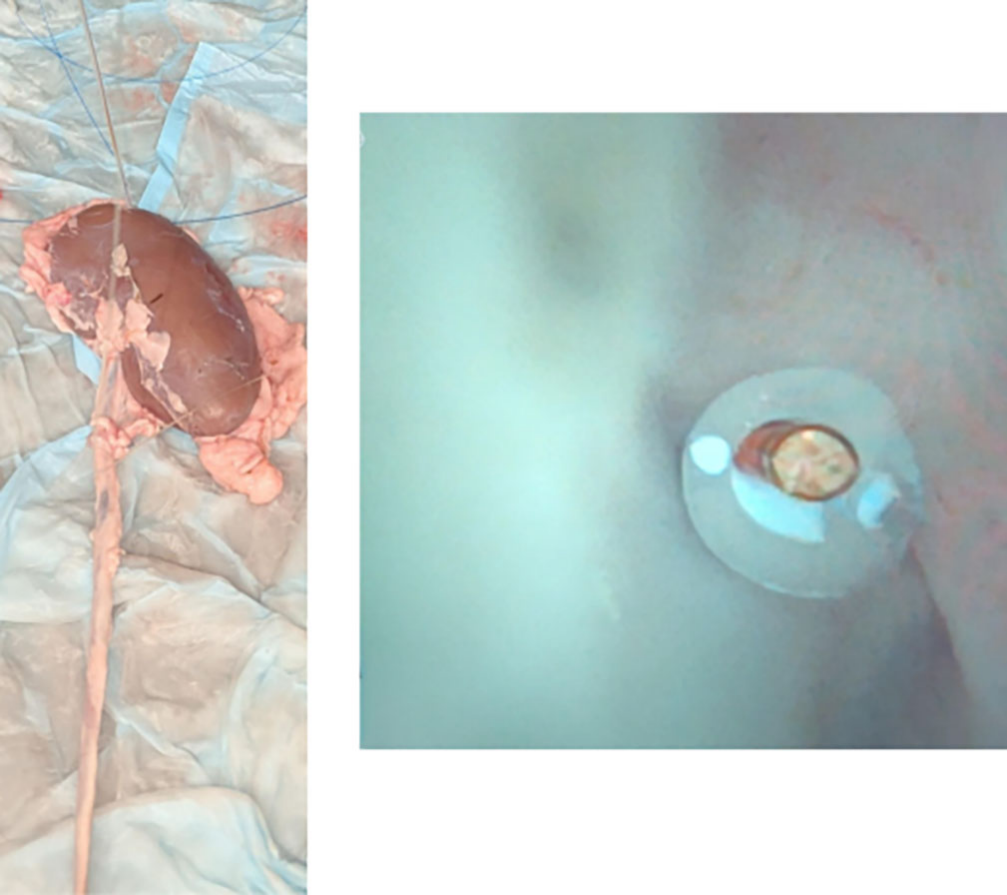
*Demonstration of Ex Vivo* porcine kidney benchtop model set‐up. Left image: Kidney and ureter with pressure sensor into the calyx and FANS in the ureter. Right image: ureteroscopic image of pressure sensor within the calyx.

We performed *ex vivo* porcine kidney experiments to study the effects of ureteroscope diameter, FANS diameter, and irrigation pressure variables on IRP and irrigation fluid flow rates (Fig. 2). The results from the *ex vivo* experiment were used to validate a mathematical model (Appendix S1) at predicting IRP and flow rates with the varying outflow resistance of varying ureteroscopes and FANS combinations [14, 16].

### *Ex vivo* Porcine Kidney Experiments

Fresh female porcine urinary tracts (warm ischaemic time <30 min) were obtained from a UK Food Standards Agency approved abattoir for food production. Within our laboratory, the ureter was cut at the bladder to facilitate insertion of FANS without trauma. Either an 11/13‐F (inner diameter = 3.7 mm) or 10/12‐F (inner diameter = 3.3 mm) 40‐cm ClearPetra*®* FANS (Well Lead Medical Co., Ltd, Guangzhou, Guangdong, China) was inserted retrograde through the ureter with its tip placed within the renal pelvis of the porcine kidney under visual guidance. The ClearPetra FANS vent remained open, with no suction applied to the FANS.

Either a 9.5 F (outer diameter = 3.2 mm) Boston Scientific LithoVue™ (Boston Scientific Corp., Marlborough, MA, USA), a 7.5‐F (outer diameter = 2.5 mm) PUSEN PU3033 (Zhuhai Pusen Medical Technology Co., Ltd, Zhuhai, Guangdong, China), or a 6.3‐F (outer diameter = 2.1 mm) HugeMed HU30M (HugeMed, Shenzhen, Guangdong, China) ureteroscope with an indwelling ‘200 μm’ SOLTIVE™ Premium SuperPulsed Laser System (Olympus Corp., Tokyo, Japan) was inserted through the sheath with its tip within a middle pole calyx.

An Amplatz Super Stiff™ wire (Boston Scientific Corp.) was used to retrogradely puncture the collecting system. An 8‐F Foley catheter was inserted antegrade over the guidewire with its tip within the calyx. The Foley catheter balloon was inflated to seal the puncture. A 2‐F fibre optic pressure sensor (FISO Technologies, Québec, QC, Canada) was inserted through the lumen of the catheter with its tip within the renal pelvis. This was used for live IRP monitoring.

Irrigation was pressurised to 0, 50, 100, 150 and 200 mmHg with a peristaltic pump (Watson‐Marlow Fluid Technology Solutions, Falmouth, Cornwall, UK) and monitored with an ‘in‐line’ Omega™ pressure transducer (PXM409, OMEGA, London, UK) in the irrigation tubing. The tubing was connected to the ureteroscope's inflow port. Irrigation pressures were maintained for 4 min. At each change of pressure, the model equilibrated for 1 min before taking the average IRP over the following 3 min. Experiments were repeated on three separate *ex vivo* porcine kidneys.

### Mathematical Model

The mathematical model has been previously described [14, 16]. A key model parameter to determine the IRP and flow rates is the outflow resistance of each combination of ureteroscope and FANS specific to the actual ClearPetra FANS sizes and scopes tested.

The outflow resistance values were derived empirically from benchtop flow rates through each combination of ureteroscope and FANS. Each of the combinations of ureteroscope and FANS were placed in an open beaker (at atmospheric pressure) of 1 L 0.9% NaCl. A fixed suction pressure was applied to the FANS (via a SAM12 medical suction device; MGE Electric Ltd, Highwoods, Colchester, UK). After 30 seconds, the change in mass of the beaker was calculated (using Ohaus Traveller™ Portable Balances; Ohaus, Greifensee, Switzerland). These flow rates at known suction pressure were used to derive the outflow resistances of each of the combinations (Table 1). For the 6.3‐ and 7.5‐F ureteroscopes, inflow resistance through the working channel with a 200‐μm laser fibre was also calculated experimentally, measuring flow rate at an irrigation pressure of 100 mmHg. Details on calculations of outflow and inflow resistances are provided in Appendix S1.

**Table 1:** Calculated outflow resistances of different combinations of ureteroscope and FANS used as a parameter in the predictive mathematical model. These resistances were calculated by aspiration of 0.9% saline through the FANS from an open beaker with 100 mmHg suction pressure.

Ureteroscope/FANS combination	Outflow resistance, mmHg/mL/min
9.5‐F ureteroscope in 11/13‐F FANS	1.90
7.5‐F ureteroscope in 10/12‐F FANS	0.83
7.5‐F ureteroscope in 11/13‐F FANS	0.39
6.3‐F ureteroscope in 10/12‐F FANS	0.29
6.3‐F ureteroscope in 11/13‐F FANS	0.18

### Modelled Scenarios

**Fig. 3:**
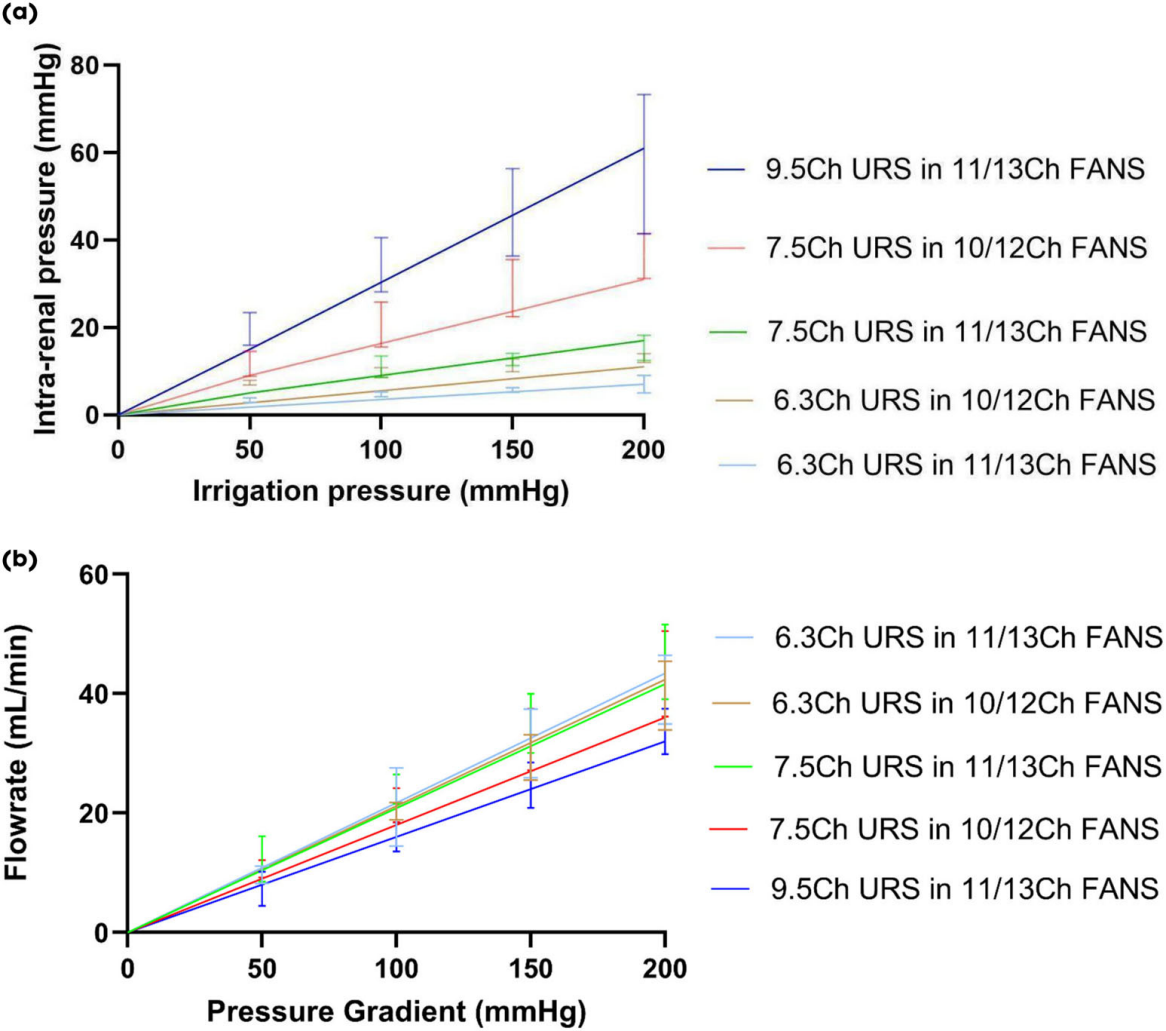
(**a, b**) Benchtop *ex vivo* porcine kidney model measured data vs theoretical mathematical model – the effect of FANS and ureteroscope size on IRP (above [**a**]) and flow rates (below [**b**]): trend lines represent model predictions, error bars represent empirically measured benchtop data 95% confidence interval ranges of three data points.

**Fig. 4:**
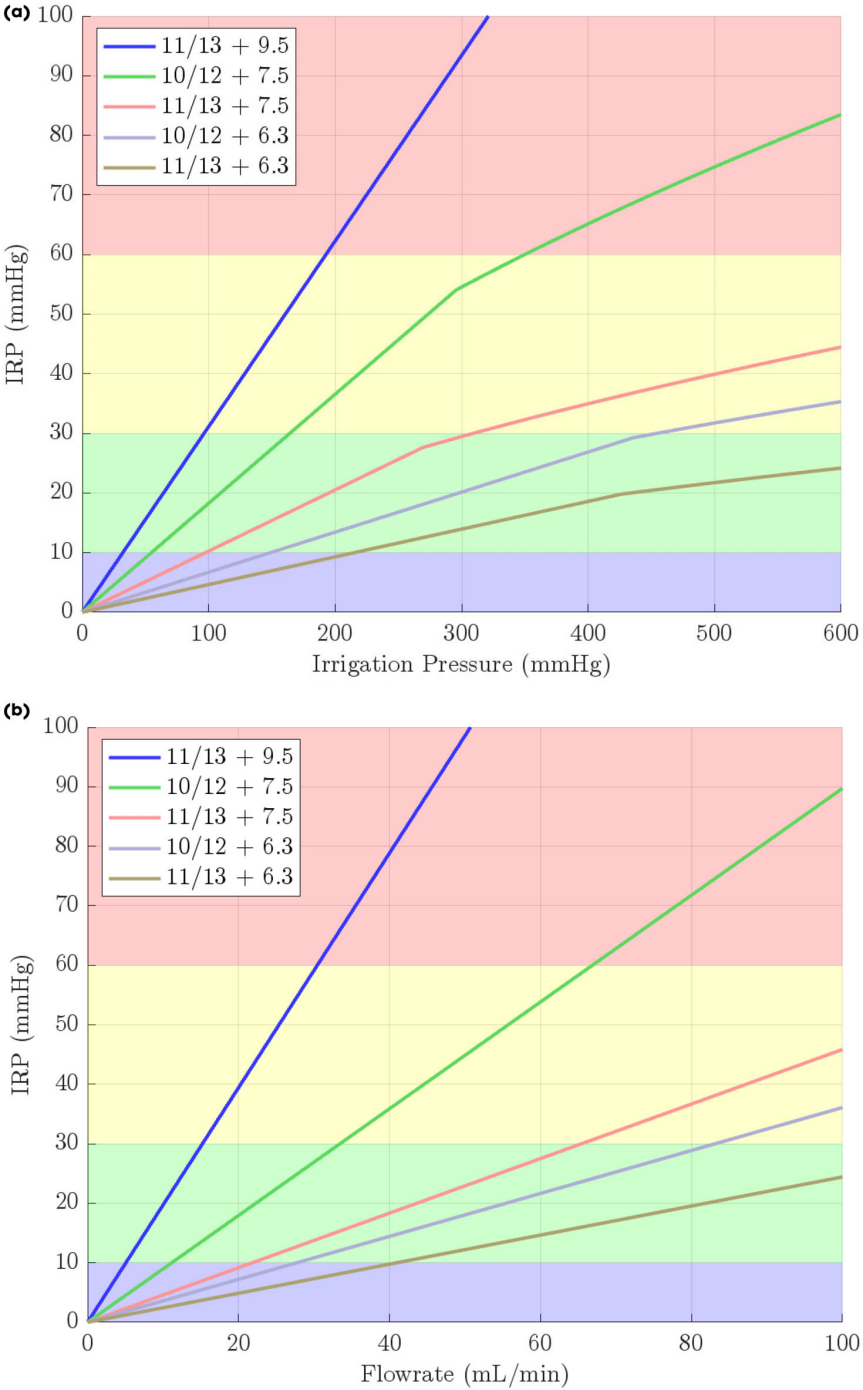
(**a, b**) Mathematical model predictions of the effect of FANS and ureteroscope size on IRP with increasing irrigation pressure (above [**a**]) and flowrates (below –[**b**]) (without the addition of active aspiration, results reflect the predictions based on the empirically derived outflow resistances of the FANS and ureteroscope combinations with the FANS tip located in the renal pelvis with varying irrigation pressure). There is a linear relationship between flow rate and IRP (**b**). Irrigation pressure vs IRP is non‐linear (**a**) as flow through the working channel changes from ‘laminar’ to ‘turbulent’ flow above a certain irrigation pressure (demonstrated as a bend in the line).

**Fig. 5:**
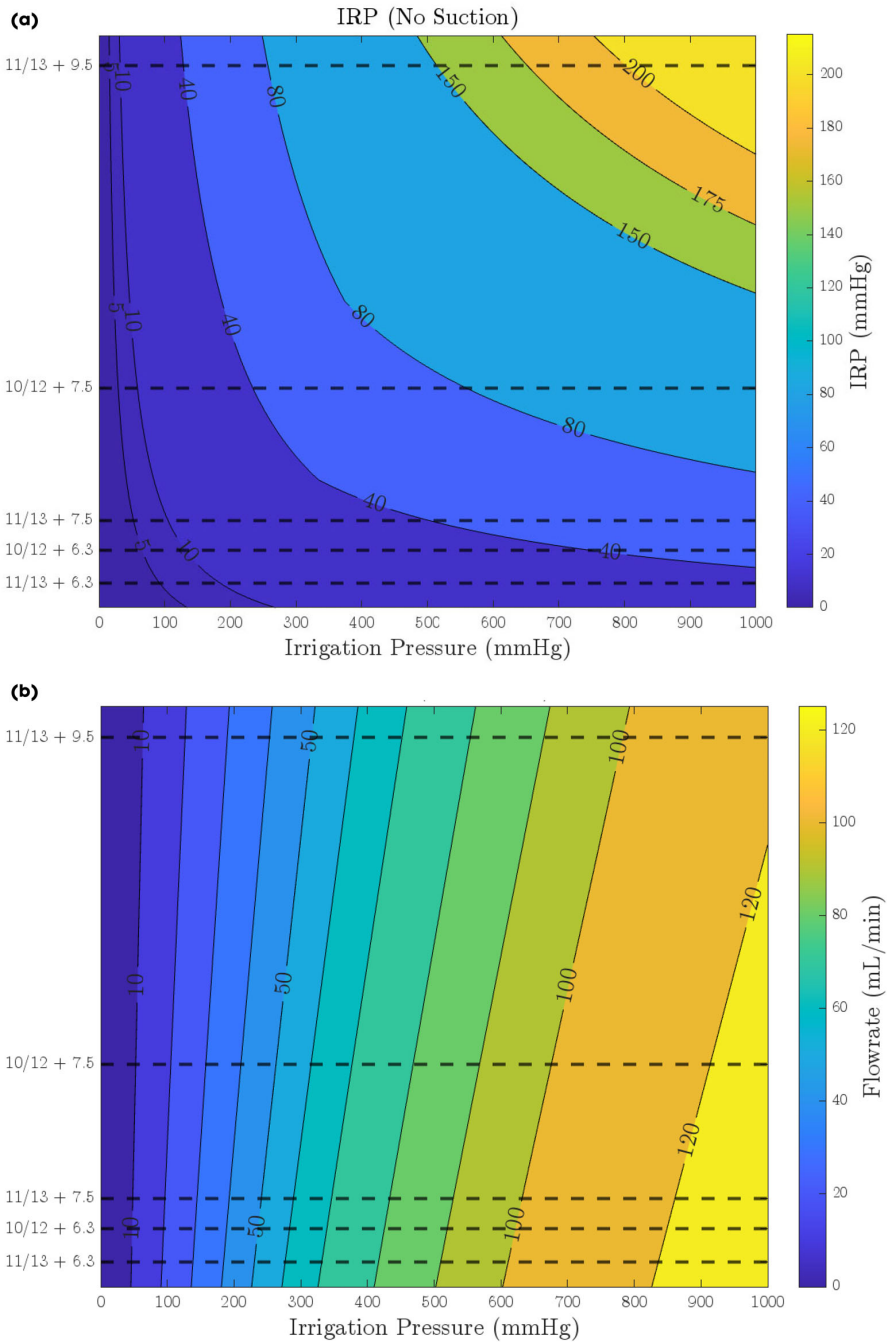
(**a, b**) Mathematical model predicted results – the effect of outflow resistance (as determined by different ureteroscope and FANS combinations) on IRP (above [**a**]) and flow rate (below [**b**]) (without the addition of active aspiration, results reflect the predictions based on the empirically derived outflow resistances of the FANS and ureteroscope combinations with the FANS tip located in the renal pelvis with varying irrigation pressure).

First, the benchtop *ex vivo* porcine kidney model measured data were plotted against the theoretical mathematical model to validate the predicted effect of FANS and ureteroscope size on IRP and flow rates (Fig. 3). Second, the maths model was extended to predict the effect of irrigation pressure and flow rates (at pressures and flow rates not tested empirically) of the different combinations of ureteroscope and FANS on IRP (Fig. 4). Third, the model was extended to predict the effect of irrigation pressure on flow rates and IRP at a range of outflow resistances (Fig. 5). Lastly, the model was extended to predict the effect of applied suction (at various pressures) with continuous irrigation, in a closed system (i.e., vent permanently closed), as a function of outflow resistance, on IRP.

## Results

The IRP depends on outflow resistance created by the geometry between the inner diameter of the FANS and the outer diameter of the ureteroscope (theoretically depicted in Fig. 1). Larger cross‐sectional spaces yield lower outflow resistance and consequently can lower IRP. The outflow resistances (as calculated by benchtop aspiration from an open beaker) of the various combinations of ureteroscope and FANS are shown in Table 1. These were used in the mathematical model to predict IRP and flow rates at fixed irrigation pressures.

The *ex vivo* porcine kidney experimental data from the FANS and scope combinations (three data points collected from each combination) and resulting IRP and flow rates measured from a given fixed irrigation pressure was plotted against the updated FANS maths model predictions (Fig. 3). There was a strong agreement between the mathematical predictive model and benchtop *ex vivo* model data in IRP resulting from different combinations of ureteroscope size, FANS size, and irrigation pressures (Fig. 3).

Both the empirical *ex vivo* kidney and theoretical maths model, demonstrate a linear increase in IRP and flow rate from increasing irrigation pressures (Fig. 3). For a fixed irrigation pressure, decreasing the outflow resistance by increasing the space between the FANS inner diameter and ureteroscope outer diameter decreases the IRP and increases the flow rate.

Experimentally the IRP was ≤30 mmHg, up to 200 mmHg irrigation pressure, with the following combinations of ureteroscope and FANS: 7.5‐F ureteroscope with 11/13‐F FANS; 6.3‐F ureteroscope with 10/12‐F FANS; 6.3‐F ureteroscope with 11/13‐F FANS (Fig. 3). Whilst the 30 mmHg threshold was breached using 100 mmHg irrigation pressure with a 9.5‐F ureteroscope in an 11/13‐F FANS and 200 mmHg irrigation pressure with a 7.5‐F ureteroscope in an 10/12‐F FANS.

Our mathematical model predicted IRP and flow rates at extended irrigation pressures not tested experimentally (Figs. 4 and 5). It once again demonstrates that by reducing outflow resistance, higher irrigation pressures and flow rates can be used whilst maintaining an IRP ≤30 mmHg (without suction) (Figs. 4, 5 and Table 2). Whilst keeping IRP ≤30 mmHg, theoretically: ~700 mmHg irrigation pressure (flow rate ~120 mL/min) could be used with a 6.3‐F ureteroscope in an 11/13‐F FANS; ~300 mmHg irrigation pressure (flow rate ~65 mL/min) could be used with a 7.5‐F ureteroscope in an 11/13‐F FANS; ~100 mmHg irrigation pressure (flow rate ~15 mL/min) could be used with a 9.5‐F ureteroscope in an 11/13‐F FANS.

**Table 2:** The minimum and maximum irrigation pressures and flow rates with different pairings of ureteroscope and FANS within a target range of 10–30 mmHg. These numbers were derived from Fig. 4.

Ureteroscope/FANS combination	Minimum irrigation pressure to achieve IRP ≥10 mmHg, mmHg	Minimum flow rate to achieve IRP ≥10 mmHg, mL/min	Maximum irrigation pressure with IRP ≤30 mmHg, mmHg	Maximum flow rate with IRP ≤30 mmHg, mL/min
6.3‐F ureteroscope and 11/13‐F FANS	~220	~40	~700	~120
6.3‐F ureteroscope and 10/12‐F FANS	~150	~30	~450	~85
7.5‐F ureteroscope and 11/13‐F FANS	~100	~20	~300	~65
7.5‐F ureteroscope and 10/12‐F FANS	~50	~10	~150	~30
9.5‐F ureteroscope and 11/13‐F FANS	~30	~5	~100	~15

Our mathematical model also predicted the IRP and flow rates needed to create an IRP of ≥10 mmHg (an estimate of the IRP needed to create operative space) (Figs. 4, 5 and Table 2). Theoretically: ~220 mmHg irrigation pressure (flow rate ~40 mL/min) would be needed with a 6.3‐F ureteroscope in an 11/13‐F FANS; ~100 mmHg irrigation pressure (flow rate ~20 mL/min) would be needed with a 7.5‐F ureteroscope in an 11/13‐F FANS; ~30 mmHg irrigation pressure (flow rate ~5 mL/min) would be needed be a 9.5‐F ureteroscope in an 11/13‐F FANS.

**Fig. 6:**
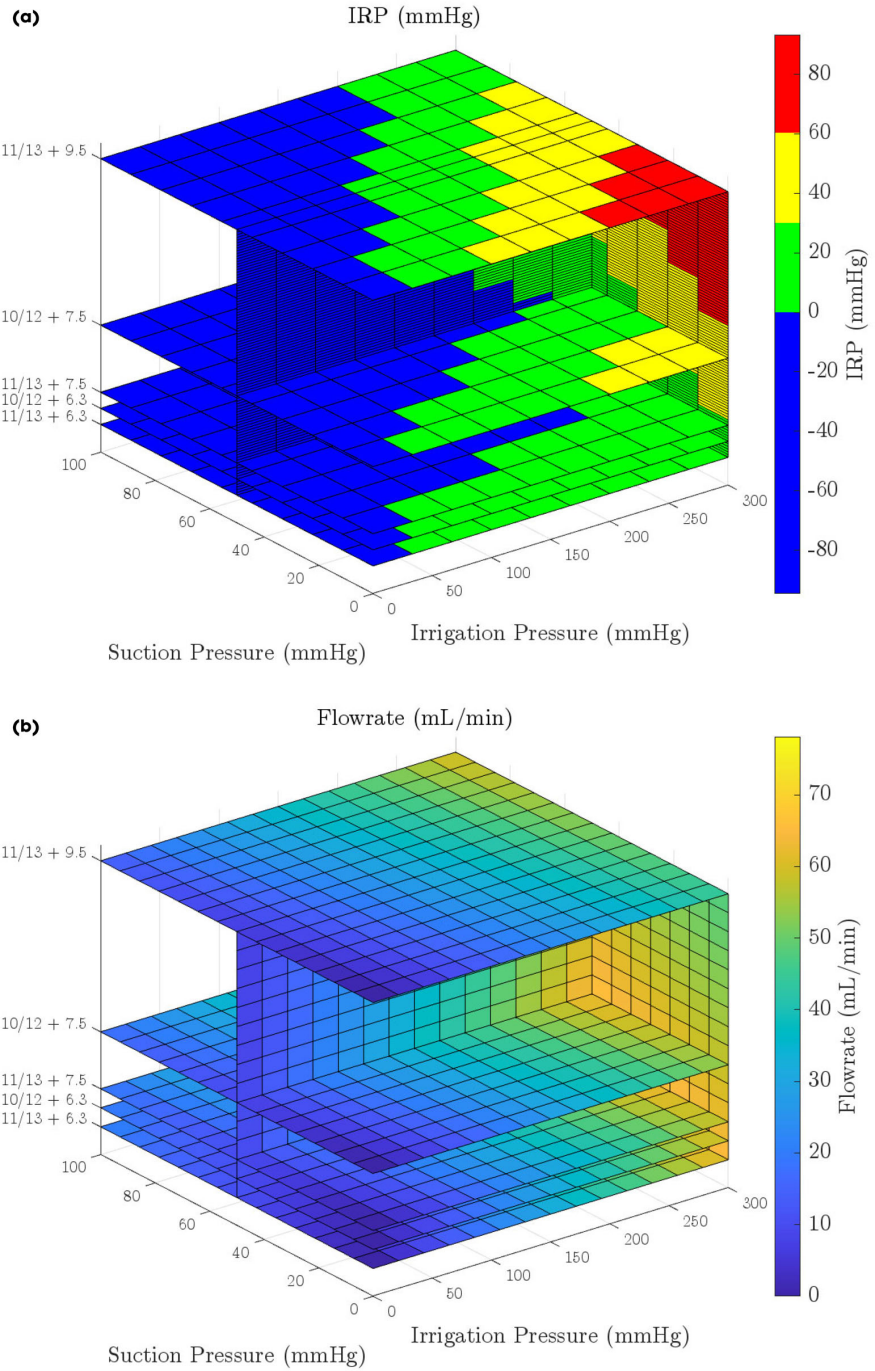
(**a**, **b**) Mathematical models showing the effect of irrigation pressure (*x*‐axis), suction pressure (*y*‐axis) and outflow resistance (*z*‐axis) on (**a**) IRP and (**b**) flow rate. In the IRP (**a**): blue = IRP ≤10 mmHg; green = IRP 10–30 mmHg; yellow = IRP 30–60 mmHg; Red = IRP >60 mmHg.

Lastly, our mathematical model predicted IRP as a function of irrigation pressure with the addition of suction pressures using FANS (Fig. 6). This is in a theoretically closed system (with the vent closed throughout the procedure), which is unusual in current practice. This demonstrates that, as outflow resistance is lower than inflow resistance, only minimal suction (negative) pressures are needed to counterbalance higher irrigation (positive) pressures. For example, with a 9.5‐F ureteroscope in an 11/13‐F FANS, an IRP of 0–20 mmHg is maintained with ~300 mmHg irrigation pressure and ~100 mmHg suction pressure. Reducing the outflow resistance (i.e., with a 7.5‐F ureteroscope in an 11/13‐F FANS) requires even lower suction (negative) pressure to maintain the same IRP range – with ~300 mmHg irrigation pressure and ~40 mmHg suction pressure.

## Discussion

Currently there is limited understanding of how irrigation pressure correlates with IRP with various combinations of ureteroscope and FANS diameters. This work characterised IRP and flow rates as a function of outflow resistance specific to the geometries of clinically available ureteroscopes where the FANS tip is placed within the collecting system. Several authors have previously attempted to define the optimal pairing of ureteroscope and ureteric access sheath diameters using either: (a) the absolute difference in size (F) between the ureteroscope and sheath, or (b) the ratio of endoscope to sheath diameter (RESD) [17, 18].

However, the same absolute difference in size (F) or RESD does not necessarily correspond to similar outflow dynamics with different combinations of ureteroscopes and FANS. For example, a 10/12‐F sheath used with a 7.5‐F ureteroscope provides an outflow cross‐sectional area of 3.8 mm^2^, whereas a 12/14‐F sheath paired with a 9.5‐F ureteroscope results in a larger area of 4.7 mm^2^, despite both combinations having the same absolute difference of 2.5 F. Similarly, although a 10/12‐F sheath with a 7.5‐F ureteroscope and a 12/14‐F sheath with a 9‐F ureteroscope both yield an RESD of 0.75, their outflow cross‐sectional areas differ markedly: 3.8 vs 5.5 mm^2^, respectively. As a result, other authors have previously taken a similar approach to us and empirically measured the outflow resistance of different traditional ureteric access sheaths [19].

Instead of relying solely on size (F) differences or RESD, our approach empirically tested outflow resistance of actual FANS and ureteroscope geometries to more directly evaluate IRP and flow rate outcomes.

Defining a range of outflow resistances that support clinically useful flow rates whilst maintaining a target IRP range would provide a more practical and physiologically relevant framework (Table 1, Fig. 5a,b). Clinically, higher flow rates can improve operative vision and may allow higher laser power to be used by mitigating the thermal dose [10]. Higher laser power increases the stone ablation rate linearly [20], potentially making retrograde intrarenal surgery (RIRS) laser lithotripsy faster and enabling the treatment of larger renal stones by RIRS. However, excessively low outflow resistance can be counterproductive. First, with a very low outflow resistance (e.g., a 6.3‐F ureteroscope in an 11/13‐F FANS) it may be difficult to maintain the minimum irrigation pressures (~220 mmHg) required to distend the collecting system (IRP ≥10 mmHg) with conventional pressure irrigation bags or gravity. Second, if these irrigation pressures are maintained, the flow rates generated (40 mL/min) may be excessively high. High flow rates could create unwanted stone movement, especially for smaller stone fragments, making them harder to precisely target when lasering.

With currently available ureteroscopes and FANS, outflow resistance can be low enough to theoretically allow flow rates of up to 120 mL/min (at IRPs ≤30 mmHg), even without the application of suction. This is 24 times higher than the flow rates often achieved with gravity irrigation at 80 cm H_2_O (~5 mL/min) [15].

Our data suggest that (without suction), a FANS/ureteroscope outflow resistance range of 0.29–0.39 mmHg/mL/min allows for relatively high maximum flow rates up to 65–85 mL/min (with an IRP ≤30 mmHg), with minimums of 20–30 mL/min (IRP ≥10 mmHg). This outflow resistance is created by the following combinations: a 7.5‐F ureteroscope with 11/13‐F FANS or a 6.3‐F ureteroscope with 10/12‐F FANS. These combinations may generate flow rate ranges likely to offer the benefits of higher flow rates useful in treating large stones whilst also allowing lower flow rates required for use in smaller stones or fragments. By contrast, an outflow resistance of 0.83 mmHg/mL/min (i.e., a 7.5‐F ureteroscope in a 10/12‐F FANS) limits the maximum flow rate to ~30 mL/min (up to an IRP of 30 mmHg), which is below the 40 mL/min previously demonstrated as potentially beneficial to mitigating laser activated thermal risk [9, 10, 11].

However, it is important to note that during URS stone debris is likely to wash into the outflow channel of FANS (between the ureteroscope outer wall and the FANS inner wall). This may collect within the outflow channel, increasing outflow resistance or blocking outflow entirely. If the outflow resistance increases (and the surgeon is unaware of it) whilst using high irrigation pressures, then this may result in raised IRPs. Thus, live IRP monitoring may be an important adjunct to use high irrigation pressures/flow rates with confidence.

Our experimental data describe IRP and flow rates in the absence of applied suction. This approach reflects the functionality of most currently available FANS, which incorporate vent designs that, when uncovered (as is typically the case), may function as an air off‐valve [14]. As a result, suction applied to the FANS may not influence IRP during the majority of the procedure; instead, ambient air may be preferentially aspirated through the open vent [14].

Future FANS designs may feature a closed vent where continuous suction is used alongside continuous irrigation pressure to reduce the IRP. Our maths model predictions when adding suction supports that currently available FANS and ureteroscopes have outflow resistance lower than inflow resistance, which suggests that only minimal suction (negative) pressures (needed to counterbalance higher irrigation [positive] pressures) would be required for beneficial flow rate and IRP ranges.

The main limitation of our study is that the experiments were performed on *ex vivo* rather than *in vivo* porcine kidneys. However, there have been several *in vivo* porcine and clinical studies demonstrating reduced IRPs with larger diameter FANS suggesting that the physics of outflow resistance is likely to hold true *in vivo* [8, 21]. A further limitation is that outflow resistance in our study was defined by using commercially available ureteroscopes of different diameters within only a ClearPetra FANS of known internal lumen. However, outflow resistance is influenced not only by outflow cross‐sectional area but also by device‐specific design characteristics such as inner wall texture and channel configuration [14]. Therefore, whilst our findings provide useful estimates of FANS of similar size (F), the precise values may not be directly transferable to all FANS models.

## Conclusion

With a FANS placed in the collecting system (proximal to the PUJ), outflow resistance is governed by the cross‐sectional area between the ureteroscope and the inner lumen of the FANS. This resistance determines the range of irrigation flow rates that can be achieved whilst maintaining a target IRP range of 10–30 mmHg.

When outflow resistance is low, e.g., using a using a small‐diameter ureteroscope within a large‐diameter FANS, it is possible (without the need for suction) to generate very high flow rates (~120 mL/min) – 24 times the flow generated from gravity irrigation – at low IRPs. Such high flow rates can help mitigate the thermal dose delivered during laser activation by dissipating heat more effectively. This may, in turn, permit the safe use of higher laser power during RIRS, potentially increasing ablation efficiency and expanding the feasibility of treating larger stones.

However, excessively low outflow resistance can be counterproductive. For example, pairing a 6.3‐F ureteroscope with an 11/13‐F FANS, requires a minimum of ~40 mL/min to create enough operative space (corresponding to an IRP ~10 mmHg). This flow rate could prove too high when treating small stones – causing excess stone movement.

Therefore, it is important to tailor the outflow resistance (ureteroscope and FANS size) to the clinical scenario. FANS and ureteroscope combinations with outflow resistance range within 0.29–0.39 mmHg/mL/min (e.g., a 7.5‐F ureteroscope with 11/13‐F FANS or 6.3‐F ureteroscope with 10/12‐F FANS) and without the use of suction can permit flow rates up to ~65–85 mL/min with an IRP ≤30 mmHg and down to ~20–30 mL/min to create enough operative space with an IRP ≥10 mmHg.

## Disclosures

Benchtop test results may not necessarily be indicative of *in vivo* clinical performance. The testing was performed by or on behalf of Boston Scientific Corp. Data on file.

## Disclosure of Interests Statement

This study was funded by a research grant from Boston Scientific Corp. Some authors involved in the study (Jessica Williams and Candace Rhodes) are employees of Boston Scientific Corp. Richard Menzies‐Wilson and Ben Turney are recipients of Boston Scientific Corp. research grant funding. Thijs Ruiken has no disclosures.

## Supporting information


**Appendix S1.** A mathematical model to predict flow rates and IRPs as a function of irrigation pressure, suction pressure, inflow resistance, and outflow resistance.
