# Supplementary material for: Flexible and navigable suction access sheaths: balancing sheath and scope size for desired flows
Source: BJU Int. 2025 Sep 26;137(Suppl 3):S60–70. doi: 10.1111/bju.16865 (PMC12950934; doi:10.1111/bju.16865)
Supplement: Supplementary file 1 — Appendix S1. A mathematical model to predict flow rates and IRPs as a function of irrigation pressure, suction pressure, inflow resistance, and outflow resistance. [file BJU-137-S60-s001.docx]

**Appendix A**

The mathematical model^13,14^ calculates flow rate and IRP as functions of irrigation pressure, suction pressure, inflow resistance, and outflow resistance, where inflow resistance and outflow resistance govern the relationship between pressure drop and flow rate through the working channel and sheath, respectively. Due to the complicated cross-sectional geometries of the working channel with a tool, and the FANS with an indwelling scope, inflow and outflow resistances were calculated experimentally and used as inputs into the model, rather than with physics-based formulas.

Inflow resistance for the 9.5 F scope and outflow resistance through the 11/13 FANS (with an indwelling 9.5 F scope) were previously calculated^14^.

Outflow resistances for all other scope and sheath combinations with no suction tubing attached were calculated using benchtop aspiration from an open beaker; these experiments applied a known pressure to the end of the FANS (either 100 mmHg or 200 mmHg) and measured the resulting flow rate through the FANS. The contribution to outflow resistance from the FANS and ureteroscope geometries was then calculated by dividing pressure by flow.

As the *ex vivo* experiments were performed with suction tubing attached the contribution to outflow resistance through the addition of suction tubing alone was measured by applying 50 mmHg suction and measuring flow rate. The suction tubing resistance values were added to each of the FANS/ureteroscope outflow resistances to determine a total outflow resistance.

Inflow resistance for the 6.3 F and 7.5 F scopes was calculated by applying 100 mmHg irrigation pressure and measuring flow rate through the scope and similarly dividing pressure by flow rate. Although all working channels are listed as 3.6 F, inflow resistances varied between scopes by at most 25%.
